# Revealing the Therapeutic Potential: Investigating the Impact of a Novel Witch Hazel Formula on Anti‐Inflammation and Antioxidation

**DOI:** 10.1111/jocd.16662

**Published:** 2024-11-22

**Authors:** Xue Liu, Tamer‐Whittle Hage, Li‐Chi Chen, Eddy Hsi Chun Wang, I‐Chien Liao, Jodi Goldberg, Sabina Gosto, Paula Cziryak, Maryanne Senna, Ying Chen, Qian Zheng

**Affiliations:** ^1^ L'Oreal Research and Innovation Clark New Jersey USA; ^2^ Harvard Medical School, Boston & Beth Israel Lahey Health Burlington Massachusetts USA

**Keywords:** anti‐inflammatory, antioxidant, barrier function

## Abstract

**Background:**

Skin barrier health is crucial for preventive and corrective skincare across all skin types. Witch hazel (*Hamamelis virginiana*) extracts show potential in addressing skin issues, but their efficacy in treating chronic inflammation, improving skin barrier function, and combating UV‐induced oxidation requires further investigation.

**Aims:**

To evaluate the efficacy of a novel formula containing witch hazel extracts in treating chronic inflammation, improving skin barrier function, and combating UV‐induced oxidation.

**Methods:**

We employed a novel ex vivo chronic inflammation model to assess anti‐inflammatory effects, measuring key pro‐inflammatory cytokines. Barrier function markers, such as loricrin and transglutaminase‐1, were analyzed. An ex vivo model with UV‐induced Reactive Oxygen Species (ROS) elevation was used to evaluate antioxidant properties, measuring specific ROS markers like 4‐Hydroxynonenal and carbonylated protein.

**Results:**

The novel witch hazel formula significantly reduced pro‐inflammatory cytokines in both 2D and ex vivo models, including IL‐6 and IL‐8, demonstrating potent anti‐inflammatory effects. Barrier function markers showed notable improvements compared to the inflamed condition. In the UV‐induced ROS model, the formula remarkably decreased ROS levels, specifically 4‐Hydroxynonenal and carbonylated protein, indicating strong antioxidant properties.

**Conclusions:**

Our findings demonstrate that the novel witch hazel formula exhibits potent anti‐inflammatory and antioxidant properties while enhancing skin barrier function. This natural, well‐tolerated ingredient offers a promising treatment option for improving overall skin health, presenting new opportunities in skincare formulation and treatment strategies.

## 1 Introduction

Witch hazel (*Hamamelis virginiana*) is a natural plant extract known for its medicinal properties for centuries. Obtained from the leaves and bark of the North American witch hazel shrub, this extract contains a complex mixture of bioactive compounds that contribute to its multifaceted benefits [1]. Tannins, the primary constituents of witch hazel extract, account for its remarkable astringent properties [2]. By tightening and toning the skin, tannins might be able to help reduce pore size, control oil production, and provide a refreshing sensation [3]. Flavonoids, another group of bioactive compounds found abundantly in witch hazel, possess noteworthy antioxidant and anti‐inflammatory effects [4]. These properties make witch hazel an excellent candidate for combating the harmful effects of free radicals and protecting the skin from oxidative stress. Inflammation, a common underlying factor in various skin conditions, can also be alleviated by the anti‐inflammatory action of witch hazel's flavonoids. This contributes to improvement of skin health.

In addition to tannins and flavonoids, witch hazel contains other polyphenolic compounds such as proanthocyanidins and catechins [5]. These compounds further enhance the biological activity of witch hazel, working in synergy with tannins and flavonoids to provide comprehensive benefits. Proanthocyanidins, known for their antioxidant properties, help neutralize harmful free radicals, while catechins offer anti‐inflammatory effects and aid in maintaining skin elasticity.

Scientific research has substantiated the positive effects of witch hazel on skin health. Studies have shown that witch hazel extract can effectively reduce inflammation associated with acne, eczema, and other skin conditions using different models [1, 4, 5, 6, 7, 8]. By soothing irritated skin and preventing bacterial growth, witch hazel can promote a clearer complexion. Additionally, its potent antioxidants combat free radicals and environmental stressors, helping to prevent premature aging and maintain skin health [4].

The multiple benefits of witch hazel have led to its widespread use in various skincare products. It is a common ingredient in toners due to its ability to balance the skin's pH level and tighten pores. Cleansers formulated with witch hazel can effectively remove impurities and excess oil, leaving the skin clean and refreshed. Additionally, witch hazel is often incorporated into moisturizers for its soothing properties and capacity to enhance the overall health and appearance of the skin [9].

The potential of witch hazel in promoting skin health warrants further investigation to fully understand its comprehensive range of benefits. In this study, multiple studies have examined the efficacy of witch hazel extracts for skin care applications. A 2D cellular model mimicking inflammation conditions and a novel inflamed ex vivo skin model was used to study the anti‐inflammatory benefits of witch hazel. We also used a UV‐induced ex vivo skin to investigate the antioxidation effect from the formula.

## 2 Materials And Methods

### 2.1 In Vitro Studies

#### 2.1.1 Cytotoxicity

In this study, we investigated the effects of witch hazel solution on HaCat cells. The cells were first plated in a 96‐well plate at a density of 13 500 cells per well in growth medium and allowed to attach overnight at 37°C with 5% CO_2_. The following day, the cells were treated with varying concentrations of the witch hazel solution, including 0.015%, 0.5%, 1%, 2%, 4%, and 8%, as well as different solvent concentrations (0.015%, 2%, and 8%), and incubated for 24 h. After incubation, the growth media was removed and replaced with media containing 1 mg/mL MTT (Thiazolyl Blue Tetrazolium Bromide, Cat#M5655, Millipore Sigma, St. Louis, MO, USA) and incubated for 2 h at 37°C with 5% CO_2_. Following this, the MTT solution was removed and replaced with 200 μL of 200pf EtOH and placed on a plate shaker. After 15 min, absorbance was read at 570 nm, and the absorbance values of the samples were averaged and compared to controls.

#### 2.1.2 Cell Culture

The HaCaT keratinocyte cell line (AddexBio, cat#T0020001 lot number 00003978) was cultured in Dulbecco's Modified Eagle Medium (DMEM) supplemented with 10% fetal bovine serum, L‐glutamine (2 mM) and penicillin/streptomycin solution (5 U/mL) purchased from ThermoFisher cat# 30‐001‐CI. Cells were maintained at 37°C and 5% CO_2_ in a humidified incubator. For experiments, cells were seeded in 12‐well plates at a density of 160 000 cells per well and allowed to attach overnight. The next day, the medium was removed, and cells were washed once with warm Dulbecco's phosphate‐buffered saline (DPBS) (Corning cat#21‐031‐CM). Cells were then treated with witch hazel solution formula at concentrations of 2%, 4%, and 8% in the presence of 20 ng/mL of TNFα (Cat#210‐TA, lot#DDHB0320022, R&D Systems, Minneapolis, MN, USA). Four biological replicates were used for each treatment condition. The cells were incubated for 24 h under standard culture conditions.

#### 2.1.3 RNA Isolation

The HaCaT cells were treated with the designated treatments for 24 h. After 24 h, the media was removed and Qiagen RLT lysis buffer containing 1% β‐mercaptoethanol was added to each well. The cells were freeze/thawed at −80°C for two times to ensure cell lysis. Total RNA was then extracted using the Qiagen RNeasy Mini Column kit (Cat #74108, Qiagen, Hilden, Germany) according to the manufacturer's instructions. RNA concentration and purity were measured using a Nanodrop spectrophotometer (ThermoFisher, cat# ND‐1000). A total of 0.17 μg of RNA was used in the reverse transcription reaction for each sample. The reverse transcription reaction was performed under the following conditions: 25°C for 10 min, 37°C for 2 h and 85°C for 5 min.

#### 2.1.4 Reverse Transcription Quantitative Polymerase Chain Reaction (RT‐qPCR)

In the RT‐qPCR analysis, 10 ng of cDNA was used per reaction, and each sample was run in four replicates using primers for IL‐1α, IL‐1β, IL‐8, and PGE2 according to the Applied Biosystems protocol. The analysis was performed using the Applied Biosystems Expression Suite Software v1.3. The data were normalized to the reference gene TBP and the resulting ΔCt values were calculated for each target gene by using the housekeeping Ct value for each respective biological replicate. The ΔCt values were averaged to obtain ΔΔCt values, which were then normalized to the appropriate untreated control average delta Ct value to determine the ΔΔCt values for each biological replicate. The RQ values were calculated based on the ΔΔCt values, and the resulting RQ values for the four biological replicates were averaged. Error bars were generated using the standard deviations of the four replicate values.

### 2.2 *Ex Vivo* Skin Model

#### 2.2.1 *Ex Vivo* Tissue Culture and Inflammatory Model

The *ex vivo* skin tissue used in the inflammatory model was obtained from fresh ex vivo skin (three donors, 41 years old, female, Caucasian; 49 years, female, Caucasian, and 52 years old, female, Caucasian) 1 day post‐abdominoplasty procedure (BioIVT, Westbury, NY, USA), with informed consent from all donors and approval from the WCG IRB (IRB tracking number: 20180798). The hypodermis layer was removed, and the tissue was washed twice with Phosphate‐buffered Saline (PBS) to remove blood residue. The tissue was then cut into 12 mm diameter biopsies and treated with or without Cell Stimulation Cocktail (CSC, eBioscience Cell Stimulation Cocktail (500×) Cat# 00‐4970‐94, Thermo Fisher Scientific, Waltham, MA, USA). For tissues representing a chronic inflammation condition, a concentration of 2× CSC solution was used in the culture media. For tissues treated with formula, 2%, 4%, and 8% of the formula were added with or without 2 × CSC, and the treatment was added systemically. All tissues were cultured at air‐liquid interface in Dulbecco's Modified Eagle's medium (650 μL per well, DMEM with 10% Fetal Bovine Serum and 1% penicillin–streptomycin) at 37°C with 5% CO_2_. Skin explants not subjected to CSC or formula treatments were used as the untreated control group. The media was changed every other day, except for weekends.

#### 2.2.2 Histological and Immunohistochemical Staining

Ex vivo skin tissues underwent hematoxylin and eosin (H&E) staining using a standard protocol at Histowiz Laboratory in Brooklyn, NY. Tissue samples were first fixed in 10% formalin and then processed and embedded in paraffin wax. For Transglutaminase‐1 (TGM‐1), immunohistochemistry was performed on a Bond Rx autostainer from Leica Biosystems (Buffalo Grove, IL, USA) with heat‐induced antigen retrieval at pH6 using standard protocols. The sections stained against TGM‐1 were incubated with anti‐TGM‐1 primary antibody (Rabbit polyclonal, 1:500 dilution, Cat# 12912‐3‐AP, ProteinTech, Rosemont, IL, USA). The primary antibody used for loricrin staining is from Abcam (Rabbit polyclonal, 1:250 dilution, Cat# ab85679, Abcam, Waltham, MA, USA), with heat‐induced antigen retrieval at pH9 using standard protocols. All sections were counterstained with a hematoxylin nuclear stain and then dehydrated and film coverslipped using a TissueTek‐Prisma and Coverslipper from Sakura. Finally, whole slide scanning (40×) was performed on an Aperio AT2 (Leica Biosystems, Buffalo Grove, IL, USA).

#### 2.2.3 Flow Cytometry‐Based Multiplex Cytokine Assay and ELISA

In this study, we collected the supernatant (the media secreted from the explants) between Day 2 and Day 4 (media over the weekend), to assess both IL‐17A and TNFα. To perform the analysis, we used the LEGENDplex Human Inflammation Panel I (BioLegend Cat# 740809, San Diego, CA) and conducted it with a BD Accuri C6 plus flow cytometer (BD Biosciences) following the instructions provided by the manufacturer.

To briefly outline the process, the supernatants were incubated with capture beads for 2 h at room temperature. After washing the beads, the detection antibodies were added and incubated for 1 h at room temperature. Following that, the SA‐PE was introduced to the samples for a 30‐min period, after which the samples were measured using the flow cytometer. The data obtained were analyzed using BioLegend's Legendplex software.

IL‐6 cytokine was evaluated by using commercial ELISA kits according to the manufacturer's instructions (IL‐6, Cat# D6050, R&D systems, Minneapolis, MN).

#### 2.2.4 *Ex Vivo* Tissue Culture and UV Treatment

Healthy Caucasian donors undergoing abdominal plastic surgery provided human skin samples after giving informed consent for the analysis of reactive oxygen species (ROS), 4‐NHE, and carbonylated level (three donors, 55 years old, female, Caucasian; 28 years old, male, Caucasian and 56 years old, female, Caucasian). Skin samples, measuring 8 × 3 mm (diameter × thickness), were maintained at an air‐liquid interface in contact with modified Williams' E medium (Thermo Fisher Scientific, Waltham, MA, USA) for up to 4 days. Six skin samples were used for each treatment, and a volume of 4 μL of 2% and 8% witch hazel formula, solvent or PBS were topically applied and repeated every day. The skin was irradiated with UVA light using the sensor‐controlled BIO‐SUN irradiation system (Vilber Lourmat, Eberhardzell, Germany) with the UV emission spectrum ranging from 320 to 400 nm and a dose of 60–65 J/cm^2^ of UVA was given to the skin samples [10].

### 2.3 Hydroxynonenal (4‐HNE) Immunohistochemistry

At the end of the study, two skin sections from six samples for each condition were used for staining and quantification purposes. We performed immunohistochemical staining using an automated immunostainer called Dako Autostainer Link 48 (Agilent Technologies, Santa Clara, CA, USA). Tissue samples were fixed in 10% formalin and subsequently processed and embedded in paraffin wax. Heat‐induced antigen retrieval was performed using Target Retrieval Solution (EnV FLEX TRS, High pH Cat#K8004, Agilent Technologies, Santa Clara, CA, USA) in a steamer (PTLink, Agilent Technologies, Santa Clara, CA, USA). Following antigen retrieval, we blocked non‐specific antibody binding by incubating the sections in a 2% BSA (Bovine Serum Albumin Cat#A1470, Sigma Aldrich, St. Louis, MO, USA) solution for 20 min. Subsequently, the sections were incubated with the primary antibody (Rabbit Polyclonal to 4‐HNE, 1:400 dilution, Cat# ab46545, Abcam, Cambridge, UK). The anti‐4‐HNE was detected and visualized using the Dako Real Detection System, Alkaline Phosphatase/RED, Rabbit/Mouse (Cat#K5005, Agilent Technologies, Santa Clara, CA, USA). Finally, all sections were counterstained with a hematoxylin nuclear stain.

The amount of the antigen presents each slide is evaluated by estimating the intensity and the distribution of the red within the epidermis (excluding the stratum corneum).

### 2.4 ROS Evaluation

To evaluate the effects of ROS, we utilized a probe called 2′‐7′dichlorofluorescin diacetate (DCFH‐DA). Prior to irradiation, skin samples were treated with either the test compounds or vehicle overnight (approximately 16 h). Following treatment, skin samples were washed and then incubated with DCFH‐DA for 30 min. After washing with PBS, skin samples were exposed to UVA. Following irradiation, skin samples were harvested, cryofixed, and cryosectioned. Two sections from each skin sample were analyzed for subsequent image acquisition and analysis. We acquired fluorescence images using an Olympus BX51 microscope and Olympus DP70 camera (Olympus, Tokyo, Japan). The images were evaluated by transforming them from RGB to the Lab* color space, and then we analyzed the L value of each pixel within the region of interest using Image‐J application (NIH, Bethesda, USA). To avoid including irregularities and agglomerates such as blood vessels, sebaceous glands, and hair follicles, the dermis area analyzed for fluorescence was carefully selected from the upper part by following the perimeter of the basal lamina to the deep dermis. The obtained value was then normalized to the dimension of the selected area.

### 2.5 Carbonylated Protein Evaluation (OxyELISA)

After trimming fat and connective tissue, frozen skin samples were weighed and minced. Three skin samples for each tested condition were pooled and processed together by homogenization in cold extraction buffer. Total cell lysates were recovered by centrifugation. Carbonylated proteins in skin samples were determined by OxyELISA (OxiSelectTM Protein Carbonyl ELISA Kit, Cell Biolabs, #STA‐310, San Diego, CA, USA) according to the manufacturer's instructions. For all the donors, six readings were recorded for the three homogenized samples.

### 2.6 Statistical Analysis

All statistical analyses were performed using GraphPad Prism software (version 9.0, GraphPad Software, San Diego, CA, USA). Data are presented as mean ± standard error of the mean (SEM) unless otherwise specified. For comparisons between multiple groups, one‐way analysis of variance (ANOVA) was employed. A *p* value < 0.05 was considered statistically significant. Graphs were generated using GraphPad Prism, with error bars representing SEM. Statistical significance is indicated in figures as follows: ns, *p* > 0.05; *, *p* ≤ 0.05; **, *p* ≤ 0.01; ***, *p* ≤ 0.001; ****, *p* ≤ 0.0001.

## 3 Results

The HaCaT model was used to investigate the effect of TNFα on the expression of various cytokines and prostaglandins, and to evaluate the potential anti‐inflammatory properties of witch hazel (WH) blends. The highest solvent (8%) solution served as the control group. Our results showed that the expression of cytokines IL‐1α, IL‐1β, IL‐8, and prostaglandin E2 (PGE2) was significantly increased by TNFα in the HaCaT model compared to the vehicle control group (*p* < 0.05). To validate the role of TNFα, a positive control TNFα inhibitor was applied, which notably reduced the expression of IL‐1α, IL‐1β, and IL‐8, but not PGE2.

**FIGURE 1:**
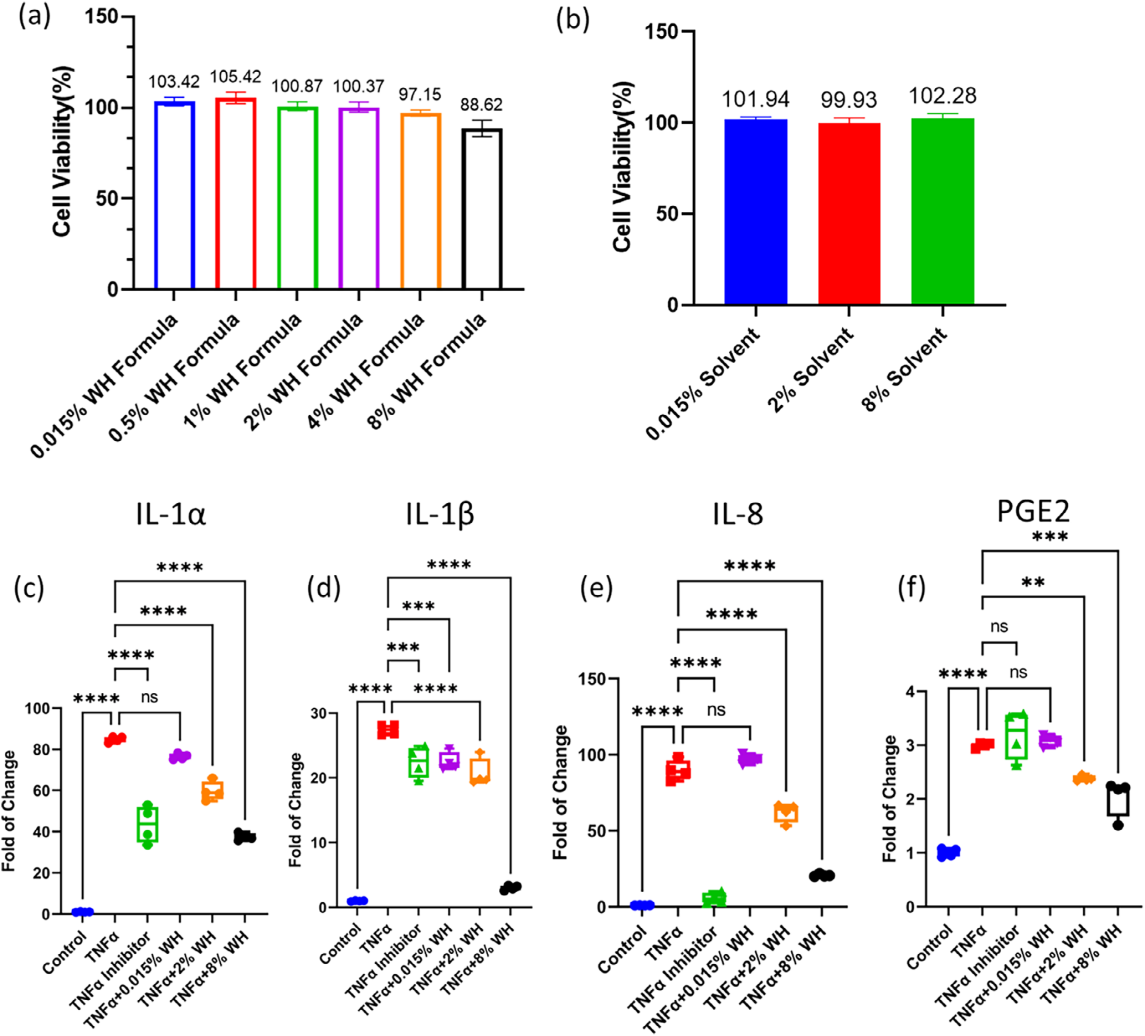
Cytotoxicity results of witch hazel blend (a) and selected relevant solvent (b). In the HaCaT model, TNFα induced significantly higher expression in IL‐1α (c), IL‐1β (d), IL‐8 (e), and PGE2 (f) compared to the control group. TNFα inhibitor (positive control) reduced the level of IL‐1α, IL‐1β, IL‐8 but not PGE2. Witch Hazel (WH) blend at concentrations of 2% and 8% reduced expression of IL‐1α, IL‐1β, IL‐8, and PGE2 significantly, while 0.015% WH blend only reduced IL‐1β significantly. All the conditions were compared to TNFα group (*N* = 3). One‐way ANOVA; ns, *p* > 0.05;  **, *p* ≤ 0.01; ***, *p* ≤ 0.001; ****, *p* ≤ 0.0001.

To evaluate the potential anti‐inflammatory effects of WH blends, different concentrations were tested. Results showed (Figure 1c–f) that 2% and 8% WH blends significantly reduced the expression of IL‐1α, IL‐1β, IL‐8, and PGE2 compared to the TNFα group (*p* ≤< 0.05). Moreover, 0.015% WH blend effectively reduced the expression of IL‐1β (*p* ≤< 0.05). These findings indicate the potential of WH blends in inhibiting the expression of various cytokines and prostaglandins, suggesting anti‐inflammatory properties.

In summary, the present study demonstrated that TNFα upregulated the expression of cytokines IL‐1α, IL‐1β, IL‐8, and PGE2 in the HaCaT model. The application of TNFα inhibitor significantly reduced the expression of IL‐1α, IL‐1β, and IL‐8, while WH blends notably inhibited the expression of all cytokines and PGE2. The results indicate the potential therapeutic application of WH blends in inflammatory skin conditions.

**FIGURE 2:**
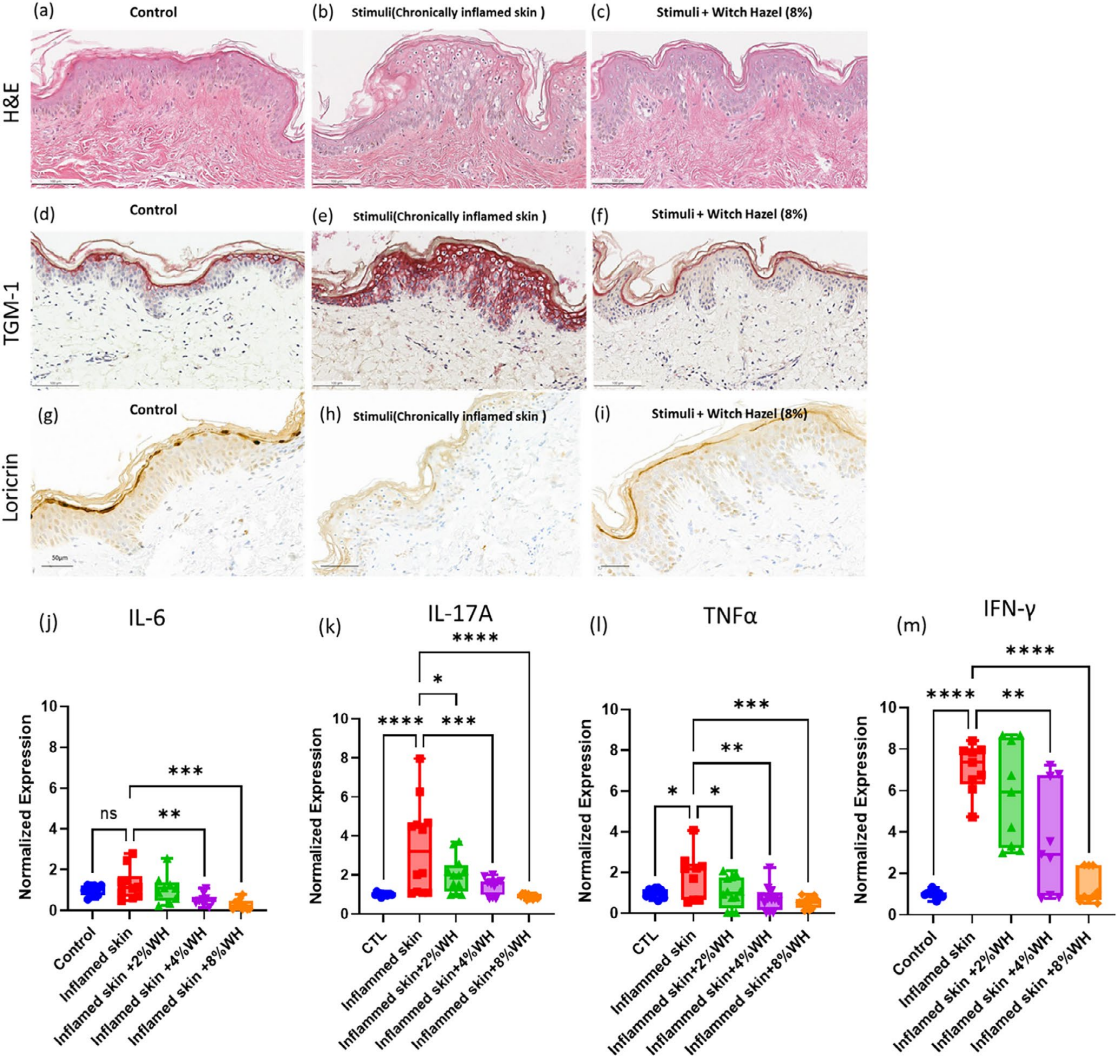
This study utilizes a novel inflammatory ex vivo skin model which exhibits several clinical hallmarks of the compromised skin, including: Spongiosis (a, b), abnormal pattern of terminal differentiation protein (TGM‐1; d, e, loricrin, g, h). Witch hazel formula showed potent efficacy in preventing the action of the stimuli in inducing inflammatory phenotype in the skin. The observed H&E morphology, TGM‐1 and loricrin expression is similar to normal control condition (c, f, i). Quantification of pro‐inflammatory cytokines in the ex vivo tissue. The expression of IL‐6 was measured with an ELISA and IL‐17A, TNFα and IFN‐γ were measured with a multiplex cytokine array (j–m). The media were collected on Day 4 (measuring the media from Day 2 to Day 4). One‐way ANOVA; *indicates *p* ≤ 0.05; ** indicates *p* ≤ 0.01; *** indicates *p* ≤ 0.001; **** indicates *p* ≤ 0.0001.

The novel inflammatory ex vivo skin model used in this study successfully replicated several clinical hallmarks of compromised skin, including spongiosis and abnormal patterns of TGM‐1 and loricrin expression. These findings validate the use of the model for discovering and evaluate potent actives research in this area [11]. The WH formula showed potent efficacy in preventing the stimuli‐induced inflammatory phenotype in the skin, as demonstrated by reduced levels of cytokines and chemokines compared to untreated skin. Moreover, the observed changes in TGM‐1 and loricrin expression in response to stimuli and WH treatment are consistent with previous studies on compromised skin conditions, further validating the use of the model in this research (Figure 2a–i). The observed H&E morphology, TGM‐1, and loricrin expression in the skin treated WH were similar to those of normal control conditions, indicating a potential restorative effect on compromised skin.

We further evaluated the cytokine changes in the tissue culture media. In the evaluation of IL‐1a levels with a single donor, the findings indicated that the vehicle did not demonstrate a significant inflammation reduction in this experimental setup (Figure S2). The expression levels of IL‐6, IL‐17A, TNFα, and IFN‐γ were evaluated with three donors. The stimulation from 2 × CSC increased the expression levels of IL‐17A, TNFα and IFN‐γ significantly (Figure 2j–m). With the treatment of 2%, 4% and 8% WH, the expression of the above‐mentioned cytokines were reduced significantly. While IL‐6 expression was not stimulated significantly with adding 2 × CSC compared to the control condition, however, with the treatment of 4% and 8% WH blend, it was reduced significantly compared to the stimulated condition. This finding indicates that WH blend have a therapeutic effect on compromised skin conditions, for example, atopic dermatitis, psoriasis, and some other prolonged inflammatory skin conditions, by reducing inflammation [12]. Furthermore, WH was found to significantly reduce the expression of IL‐6, IL‐17A, TNFα, and IFN‐γ compared to the control group in non‐stimulated conditions (Figure S1). This suggests that WH has a basal anti‐inflammatory effect on the skin, even in mild inflamed skin condition.

Overall, the results of this study suggest that the WH formula has potential as a natural anti‐inflammatory agent for compromised skin conditions. These findings have important implications for the development of new treatments for inflammatory skin conditions.

**FIGURE 3:**
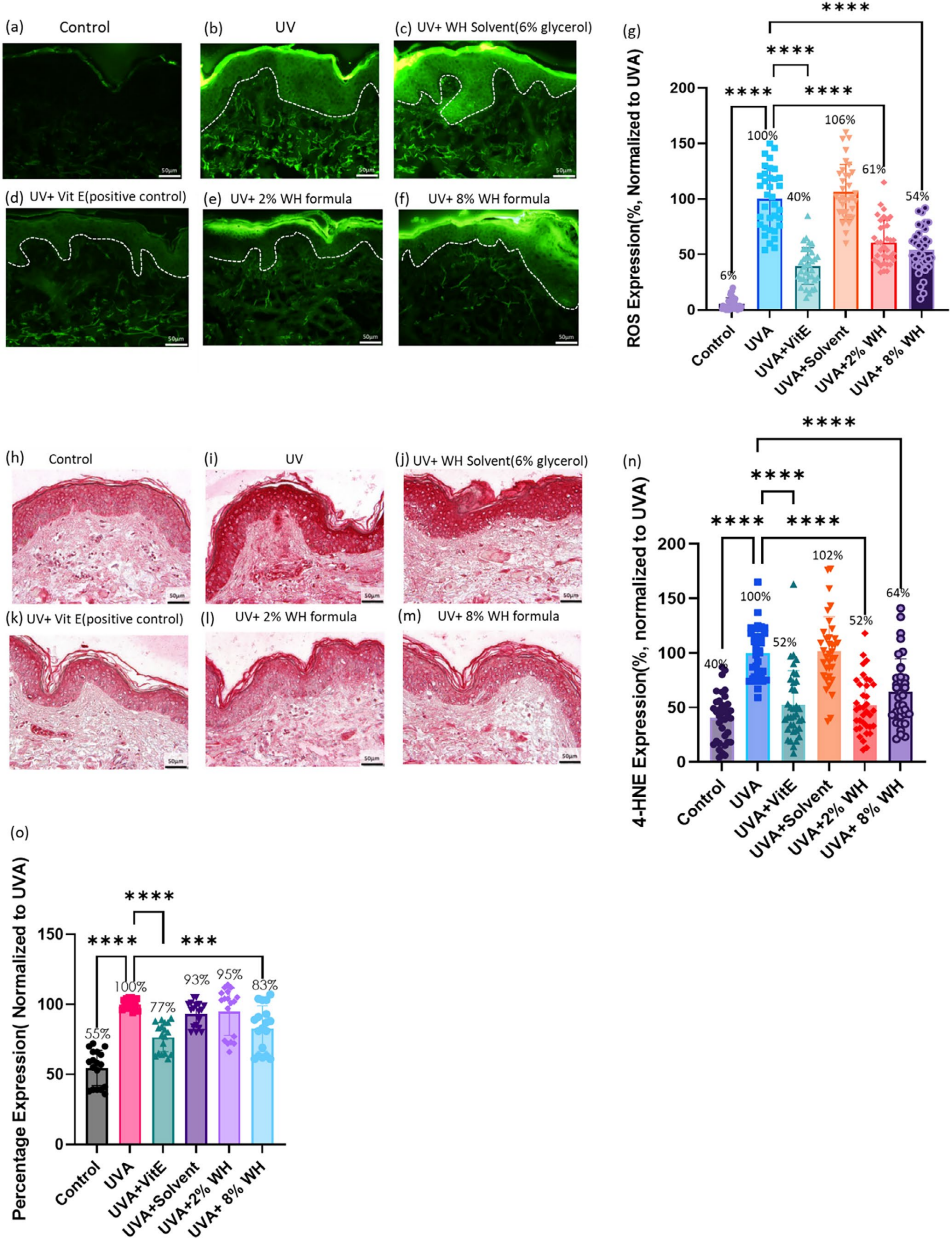
The study employed ex vivo skin to assess the antioxidation effect of the Witch Hazel (WH) formula triggered by UVA. The ROS level was measured in the samples fixed at the end of the experiments (a–g). The presence of 4‐hydroxynonenal (4‐HNE) was assessed, and it was evaluated and quantified (h–n). Additionally, carbonylated protein was evaluated by using the homogenized tissue with OyxELISA (o). One‐way ANOVA; *** indicates *p* ≤ 0.001; **** indicates *p* ≤ 0.0001.

In our study, we investigated the potential of UVA radiation to cause oxidative stress in ex vivo human skin samples of three different donors. To do so, we exposed skin samples to 60–65 J/cm^2^ of UVA and assessed ROS production using the DCFH‐DA assay. As illustrated in Figure 3, our findings revealed that UVA treatment significantly induced oxidative stress in all tissue samples.

To evaluate the efficacy of WH formula in mitigating UVA‐induced oxidative stress, we first treated skin samples topically with formula overnight (for approximately 16 h) and then exposed them to UVA. Our results indicated a significant reduction in ROS production with the use of 2% (39% reduction) and 8% (46% reduction) witch hazel formulations, compared to the ROS production of the UVA‐exposed tissues without any treatment. Moreover, our positive antioxidant control (vitamin E) confirmed the antioxidant effect in our model (60% reduction, as shown in Figure 3g). However, we observed no significant effect of the solvent in the formula in counteracting UVA‐induced oxidative stress (6% increase).

The oxidation byproducts of sebum and keratinocyte cell membrane lipid moieties play a critical role in the inflammatory response induced by UV irradiation. Among these byproducts, 4‐hydroxynonenal (4‐HNE) is one of the most extensively studied [13]. Tissue samples were collected on Days 1, 2, and 3 following UVA treatment and were used to assess 4‐HNE levels. As shown in Figure 3n, UVA exposure significantly increased 4‐HNE expression in the tissue. However, treatment with WH formula resulted in a significant reduction in 4‐HNE expression (48% and 36% reduction for 2% and 8% WH formula, respectively), indicating the potent efficacy of the WH formula in preventing and protecting against UVA‐induced 4‐HNE elevation. The biological effects of 4‐HNE are mainly attributed to covalent modification of crucial biomolecules, including proteins, DNA, and phospholipids that contain an amino group [14].

Moreover, oxidative stress can lead to protein modification, resulting in the formation of carbonyl groups. The level of protein carbonylation serves as a critical indicator for determining oxidative stress. In this study, we assessed protein carbonylation by removing the fat layer and homogenizing the tissue. As depicted in Figure 3o, UVA exposure significantly induced protein carbonylation. Figure 3o, demonstrates that witch hazel (8%) application effectively mitigated the UVA‐induced protein carbonylation level. Our results suggest that the high dosage of the WH formula was successful in counteracting the oxidative stress‐mediated increase in protein carbonylation. Overall, the experiments we carried out in this research showed the benefits of witch hazel formula on anti‐inflammation and antioxidant. This consists with the published data on tannins [15].

## 4 Discussion

Inflammation is a complex biological response of the immune system to tissue injury or infection, and it plays a critical role in the pathogenesis of many skin conditions, including acne, rosacea, psoriasis, and atopic dermatitis [16, 17, 18, 19, 20]. Inflammatory skin conditions are characterized by redness, swelling, and itching, and they can be both uncomfortable and unsightly. Anti‐inflammatory cosmetic actives such as green tea extract, niacinamide, witch hazel, aloe vera, and so forth. have been used in skincare products [4]. Based on the results obtained from the studies discussed, the novel witch hazel‐based formula has shown significant potential benefits in skincare.

In the HaCaT model, TNFα increased the expression of cytokines IL‐1α, IL‐1β, IL‐8, and PGE2, however, treatment with witch hazel formula notably inhibited the expression of all cytokines and PGE2, which correlates to the hyperpigmentation. Moreover, WH formula was found to significantly reduce the expression of multiple cytokines in a stimulated inflammatory skin model which mimics chronic inflammation skin condition. The abnormally expressed differentiation protein typically seen in patients who suffers from chronic skin inflammation problem with compromised skin barrier function was elevated in the stimulated skin groups, and the treatment with WH significantly changed the expression of the proteins and brought it back to more of normal status. These findings suggest that witch hazel‐based formula may have a therapeutic effect on compromised skin conditions by reducing inflammation.

Skin cells are constantly exposed to environmental factors such as UV radiation, pollution, and other stressors that can generate ROS and cause oxidative stress [21, 22]. Over time, this oxidative stress can lead to skin aging, wrinkles, and other signs of skin damage [21]. The antioxidant properties of WH have been demonstrated in various studies. In our ex vivo human skin samples, UVA exposure induced oxidative stress, but treatment with WH formula resulted in a significant reduction in ROS production. This finding aligns with previous research by Thring et al. (2011) [4], who reported a significant reduction in oxidative stress when WH was applied to H_2_O_2_‐treated 2D fibroblasts. While the experimental models differ—our ex vivo skin samples versus their in vitro fibroblast culture—both studies consistently demonstrate WH's ability to mitigate oxidative stress. These parallel findings across different experimental systems underscore WH's potential as an effective antioxidant agent in skincare.

Furthermore, treatment with WH formula effectively mitigated the UVA‐induced protein carbonylation level, indicating its potential as an antioxidant agent in skincare. Overall, our findings suggest that a formula with WH could be an effective tool for preventing and reducing the effects of oxidative stress on the skin and improving skin health.

We acknowledge the limited age distribution in our study, with most samples from middle‐aged individuals. Skin physiology and response to external factors vary with age, potentially affecting the efficacy of WH extract across different age groups. Future studies should include a broader age range to elucidate any age‐dependent variations in WH extract's effects. This would enhance our understanding of its mechanisms and guide its application across different age demographics.

Overall, the results from these studies suggest that the novel WH formula has significant benefits in skincare due to its anti‐inflammatory and antioxidant properties. It has the potential to reduce inflammation in compromised skin conditions and protect the skin from oxidative stress. Additionally, its ability to improve skin hydration and barrier function makes it a promising ingredient in skincare products. These findings provide a strong foundation for the development of new treatments for a range of skin conditions.

Future studies should focus on elucidating its mechanisms of action, assessing its effectiveness in treating specific skin conditions, and exploring potential synergistic effects with other natural compounds or skincare ingredients. By expanding our knowledge of WH, we can utilize its advantageous properties to develop innovative skincare solutions that cater to the diverse needs of individuals seeking optimal skin health.

## Author Contributions

Xue Liu conceptualized the research question and objectives, designed the experimental protocols, did the experiment, collected the data, analyzed the research data using statistical methods, and wrote and polished the paper. Tamer‐Whittle Hage assisted in designing the experimental setup and protocols, and carried out the experiments and collected research data. Li‐Chi Chen performed advanced statistical analyses on the research data, assisted in interpreting the results and drawing conclusions, and provided critical feedback on the manuscript. Eddy Hsi Chun Wang assisted in interpreting the results and drawing conclusions, and provided critical feedback on the manuscript. I‐Chien Liao, Ying Chen, and Qian Zheng developed the research question and objectives, helped on experimental designs, assisted in interpreting the results and drawing conclusions, and provided critical feedback on the manuscript. Jodi Goldberg, Sabina Gosto, and Paula Cziryak. developed the research question and objectives, and assisted in interpreting the results and drawing conclusions. Maryanne Senna oversaw the research project and provided guidance to the team, reviewed and revised the manuscript for intellectual content. All authors have read and approved the final manuscript.

## Conflicts of Interest

Dr. Maryanne Senna has received honoraria for consultancy from L'Oreal Research and Innovation. The remaining authors disclose no conflicts of interest. This work is fully funded by L'Oreal Research and Innovation group.

## Supporting information


Data S1.

## Data Availability

The data that support the findings of this study are available from the corresponding author upon reasonable request.
