# Supplementary material for: Revealing the Therapeutic Potential: Investigating the Impact of a Novel Witch Hazel Formula on Anti‐Inflammation and Antioxidation
Source: J Cosmet Dermatol. 2024 Nov 22;24(2):e16662. doi: 10.1111/jocd.16662 (PMC11845955; doi:10.1111/jocd.16662)
Supplement: Supplementary file 1 — Data S1. [file JOCD-24-e16662-s001.docx]

**Supplementary Data**


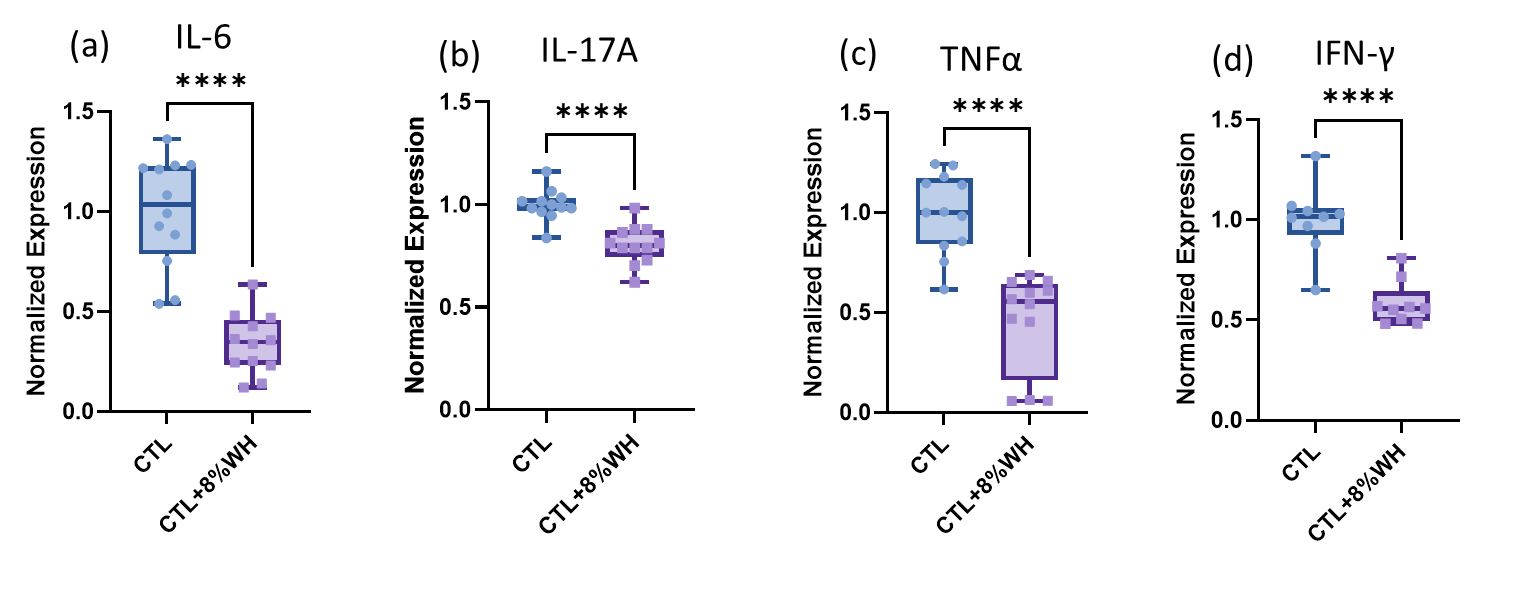


Figure S1. *Ex vivo* skin tissue with the treatment of 8% witch hazel (WH) formula significantly reduced the expression level of IL-6, IL-17A,TNFα and IFN-γ compared to the control tissues.

Figure S2. The *ex vivo* skin tissue treated with 6% glycerol did not exhibit a significant reduction in the expression of IL-1a when compared to the tissue treated with CSC.
